# How to Promote the Idea of Transplantation—Second Life Social Campaign as an Example of Successful Action in Poland—What Youth Is Used to, Adults Remember

**DOI:** 10.3390/healthcare13101203

**Published:** 2025-05-21

**Authors:** Jacek Zawierucha, Julia Piasecka, Agnieszka Patelka, Sławomir Jerzy Małyszko, Jacek Stanisław Małyszko, Jolanta Małyszko

**Affiliations:** 1 Fresenius Medical Care Polska S.A., 60-118 Poznan, Poland; jacek.zawierucha@freseniusmedicalcare.com (J.Z.); agnieszka.patelka@freseniusmedicalcare.com (A.P.); 2 Faculty of Medicine, Pomeranian Medical University, 70-204 Szczecin, Poland; julia@gradatim-sympozja.pl; 3 Department of Invasive Cardiology, University Teaching Hospital, 15-276 Bialystok, Poland; smalyszko1999@gmail.com; 4 Department of Nephrology, Transplantation and Internal Diseases, Medical University of Bialystok, 15-540 Bialystok, Poland; jackmaly@poczta.onet.pl; 5 Department of Nephrology, Dialysis and Internal Diseases, Medical University of Warsaw, 02-097 Warsaw, Poland

**Keywords:** organ donation, solid organ transplantation, social awareness

## Abstract

**Background**: Solid organ transplantation, which began as an experimental procedure over 100 years ago, has now become a viable therapeutic option for the vast majority of patients with end-stage organ failure. However, a major limiting factor remains the shortage of organs available for transplantation. To expand the donor pool, it is essential to increase awareness of transplantation, including its legal, cultural, and ethical aspects. Every year, numerous organizations, primarily non-governmental, organize hundreds of campaigns promoting transplantation worldwide. The majority of these initiatives are one-time events tied to observances such as World Transplant Day, World Kidney Day, and others. The growing role of social media in spreading information is also becoming a key factor in promoting organ donation and transplantation, as social factors play an important role in expanding the donor pool. Many organizations that support transplantation, along with healthcare providers, widely use platforms like Facebook and other social media to promote and increase organ donation. **Objectives**: The “Second Life” is a social campaign presentation to raise awarenes about transplantation. **Project Description**: The campaign has been running continuously for 17 years in Poland, promoting the concepts of organ donation and transplantation in collaboration with high school students. During this time, 950 high schools across the country participated in the project, with approximately 450,000 high school students actively engaging in the campaign. **Outcomes**: They organized a series of activities to promote organ donation and transplantation within their social circles, distributing nearly 1.5 million declarations of will. Additionally, they helped raise awareness about presumed consent and educated others on the concepts of organ donation and transplantation. **Conclusions/Implications**: This long-term initiative is crucial for raising awareness, educating the public, and promoting the idea of organ donation. It fosters a more positive attitude toward organ donation and transplantation, ultimately contributing to the expansion of the donor pool and an increase in transplantations in the near future.

## 1. Introduction

Solid organ transplantation began over 100 years ago as an experimental procedure and has since evolved into a viable therapeutic option for the vast majority of individuals with end-stage organ failure. According to data from the Global Observatory on Donation and Transplantation (World Health Organization, 2022), a total of 157,494 organ transplants were performed globally, reflecting a 9% increase in transplantations. However, the same report notes that this figure only meets 10% of the global need for transplants [1].

In Poland, 1897 solid organ transplants were performed in 2023 (50.58 per million population), and 2197 (59.38 per million population) in 2022. Despite these efforts, Poland continues to have a relatively low number of transplants compared to other countries in the European Union [2]. In addition to infrastructural and systemic challenges, such as limited bed availability, difficulties in patient qualification and organ collection, and the financial constraints of healthcare providers, the most significant barrier to increasing transplant rates remains the shortage of organs available for transplantation.

Several factors, including social, religious, psychological, and cultural elements, influence the availability of organs for transplantation. Among these, social factors play a particularly pivotal role. Thus, one key strategy for increasing the donor pool is to raise awareness of organ donation and transplantation, addressing not only its medical aspects but also its legal, ethical, and cultural dimensions.

Every year, numerous organizations—primarily non-governmental—organize hundreds of promotional campaigns worldwide. Some of these campaigns focus on specific topics, such as recruiting potential stem cell donors, while others take a broader approach, promoting the concept of organ transplantation in general. The majority of these initiatives are one-time events, often tied to global observances like World Transplant Day, World Kidney Day, and similar occasions.

The growing influence of social media in disseminating information has become increasingly important in promoting organ donation and transplantation. Many organizations that advocate for transplantation, as well as healthcare providers, actively use platforms like Facebook and other social media to raise awareness and encourage organ donation. While most of these campaigns are created with positive intentions and adhere to ethical and legal standards, the uncontrolled proliferation of such activities can unintentionally undermine the transplant system, affect potential donors, and impact transplant candidates [3]. Nevertheless, media campaigns on social media hold significant potential to effectively promote organ donation.

Brzezinski and Klikowicz [4] reported on their 5-year experience with a Facebook networking campaign, Dawca.pl Club, which garnered over 48,000 members, all of whom expressed their willingness to donate their organs after death. The authors highlighted that the mission of the campaign was twofold: to raise awareness and promote education regarding organ donation and transplantation. Additionally, their goal was to increase public acceptance of transplantation as a treatment for end-stage organ failure and expand the donor pool, both living and deceased. They used Facebook as a communication platform and emphasized that successful engagement in public health and medicine requires two-way interactions within social media. However, they also acknowledged the uncertainty about how such campaigns, despite raising awareness and acceptance, would directly translate into an increased number of donors and transplants.

Similarly, Aykas et al. [5] evaluated the influence of mass media on public opinions toward organ donation and transplantation, as well as the role and impact of public campaigns and social media in promoting organ donation. Through a systematic review of relevant literature and national campaigns, they found that, regardless of geographic region (Eastern vs. Western societies), organ donation is negatively affected by misinformation about brain death and organ transplantation. Public campaigns were found to generate only a 5% transient increase in organ donation, while the impact of Facebook was expected to be significantly higher.

More recently, Jiang et al. [6] analyzed media posts on organ donation on Weibo, a popular Chinese social media platform. They randomly extracted 141 distinct media reposts on organ donation from the posts of 16 million social media users. Their findings revealed that “statistical descriptions of organ donation” and the “meaningfulness of donation” were the most influential themes in raising awareness. Moreover, posts that focused on the “meaningfulness of organ donation for society” were particularly effective in evoking support and encouraging donation intentions. The study concluded that, in the modern world, social media plays a vital role in promoting prosocial health behaviors. They also stressed the importance of strategic message design to enhance the effectiveness of these campaigns.

Taking all of these findings into consideration, we aim to explore the effects of the “Second Life” campaign, which has been running continuously for 17 years in Poland. This initiative promotes organ donation and transplantation in collaboration with high school students, contributing to the broader effort of raising awareness and expanding the donor pool.

## 2. Transplant Landscape in the Country

The history of transplantation in Poland began in the 1960s. In January 1966, the first kidney transplant from a deceased donor was performed, followed by the first living donor kidney transplant 2 months later. However, the true breakthrough occurred in the 1980s with the introduction of new immunosuppressive agents, which led to significant improvements in transplantation outcomes. Alongside the development of transplant procedures, a framework of systems and regulations governing transplantation was also established.

Over time, the system, procedures, and regulations have evolved to meet modern legal and ethical standards, as well as the expectations of healthcare providers, patients, and payers. Today, both solid organ and stem cell transplants are performed by public healthcare providers and are fully funded by the National Health Fund (NFZ), the public payer. The NFZ covers all costs associated with recipient qualification, organ or cell collection, surgery, and post-transplant care. The majority of organs are collected from deceased donors, with only 101 (5.3%) living donor transplants performed in 2023 and 116 (5.4%) in 2024. Organ donation can only be considered from a patient declared brain dead.

Organ donation is an altruistic act based on presumed consent. According to a regulation by the Polish Ministry of Health in 1995, the Central Register of Objections to deceased organ, tissue, and cell donation was established, managed by the Polish Transplant Coordinating Center, Poltransplant. As of December 2023, the register contained 44,216 entries. The number of objections is extremely low, representing only 0.08% of the Polish population, and this rate has remained stable over time, with approximately 1000–2000 new records added annually [2,7]. Despite the presumed consent regulations and the Central Register of Objections, medical personnel typically consult with the family of potential donors regarding their wishes, and the family’s decision is given considerable weight and respect. A Declaration of Will, an informal document signed by a potential donor, is one way to express the intent to donate organs for transplantation. This document can be helpful in discussions with the donor’s family, confirming that the donation aligns with the individual’s wishes.

In 2023, 22 transplant centers performed 1897 organ transplants, and in 2024, this number increased to 2197. Additionally, in 2023, 790 allogeneic stem cell transplants were carried out in 13 hematology and stem cell transplantation centers across the country [2].

The general public’s perception of transplantation in Poland is largely positive. According to a study conducted by the Public Opinion Research Center (CBOS), 80% of respondents indicated a willingness to donate their organs after death. However, the same study revealed that 75% of respondents had never discussed their organ donation wishes with their family members. Despite the positive attitude toward organ donation, knowledge about the procedures and regulations surrounding transplantation remains moderate. While 20% of respondents were aware of the presumed consent system, 80% expressed acceptance of organ collection upon their death [8].

## 3. The Idea of Campaign

The campaign targets young people, specifically secondary school students across the country, with the goal of providing them with clear, accessible information about organ donation. It aims to encourage teenagers to engage in conversations about transplantation with their families, schoolmates, and neighbors.

**Figure 1:**
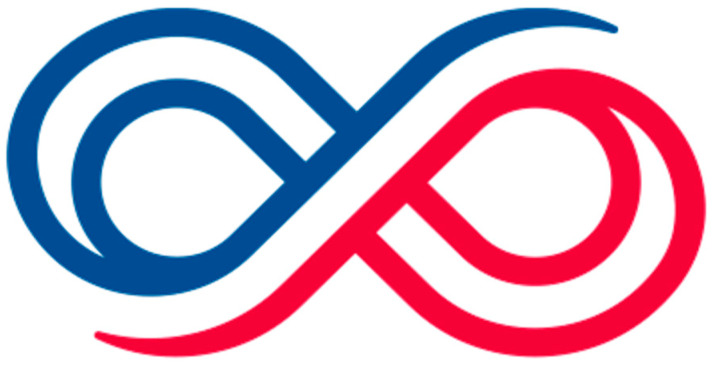
Second life campaign logo (source: www.drugiezycie.eu).

The campaign logo, designed by the PR Agency Perpetuum Media, symbolizes the continuity of life for patients after transplantation. The blue and red colors represent the organs of the donor and recipient, enabling the recipient to continue living (Figure 1).

The campaign encourages students to take a creative and proactive approach to promoting organ donation. To keep them motivated and involved throughout the school year, the campaign is structured as a competition among around 30 schools annually. Students actively share knowledge about organ donation and transplantation both online and through various events at their schools and in their local communities.

The school that generates the most engagement and spreads the message to the greatest number of people wins a monetary prize, which is donated to someone in need, chosen by the students. This encourages students to embrace the value of helping others in various ways. This campaign demonstrates that, with the right balance of expert guidance and the creativity of young people utilizing communication trends, the challenging topic of organ donation can become a natural part of everyday conversations, regardless of age, education, or religion. Encouraging open discussions about transplantation is an essential step toward making informed decisions about organ donation or understanding the views of family members on the matter. This ultimately plays a crucial role in increasing the number of transplants performed.

## 4. How It Works

Fresenius Medical Care, the organizer of the campaign, opens registration for schools at the start of each academic year. The first 30 schools to register are selected to participate in the 9-month campaign. If there are more schools interested in joining, the organizer allows Facebook users to vote for two additional schools to be included.

**Figure 2:**
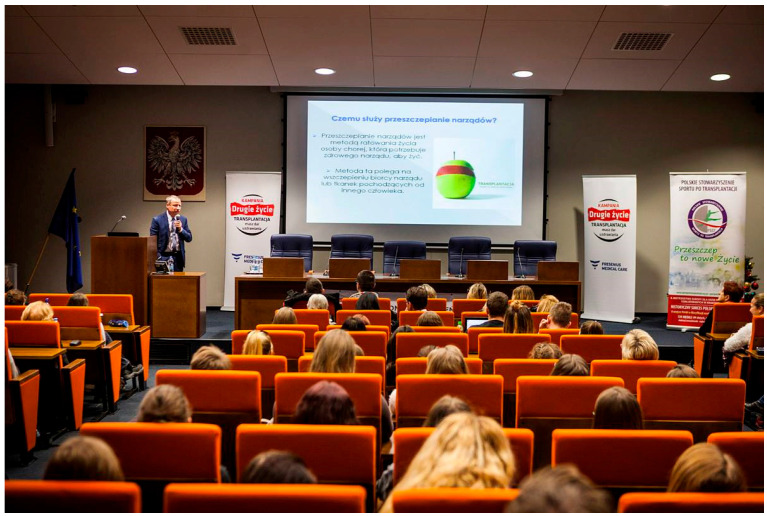
Medical training “Basics of transplantation” during XVI campaign edition launch (source: www.drugiezycie.eu).

Students from all participating schools receive comprehensive training on various aspects of transplantation. This includes medical training from transplant experts (physicians and nurses), legal training from a lawyer, and ethical guidance, typically provided by clergy. The training emphasizes presumed consent for organ donation and the significance of the declaration of intent, a valuable document in discussions with the potential donor’s family (Figure 2).

The organizer provides essential campaign materials, such as T-shirts, declarations of intent, and dedicated banners, to support the students’ efforts. The events organized by the students, in collaboration with their schools and local authorities, are designed to be low-cost or free of charge.

A key component of the campaign involves transplant recipients, who share their personal stories of receiving a new organ and gaining a “second life”. Their emotional accounts deeply resonate with the students, helping them understand that anyone can need a transplant at any time, and that promoting organ donation can change lives. The training sessions are conducted online, allowing a large number of students to participate or watch the sessions in their schools or local conference venues. These events are often attended by local authorities and enhanced with presentations by the students. This involvement attracts media attention, providing the campaign with additional, unpaid publicity. The core knowledge of transplantation enables students to share what they have learned with others. The method and channel of communication with their local communities are up to them. Participants not only share the declaration of intent during the campaign but also encourage others to sign it and keep it readily accessible, such as in their wallets.

Table 1 presents the milestones of each campaign and the initiatives carried out by students throughout the campaign cycle.

**Figure 3:**
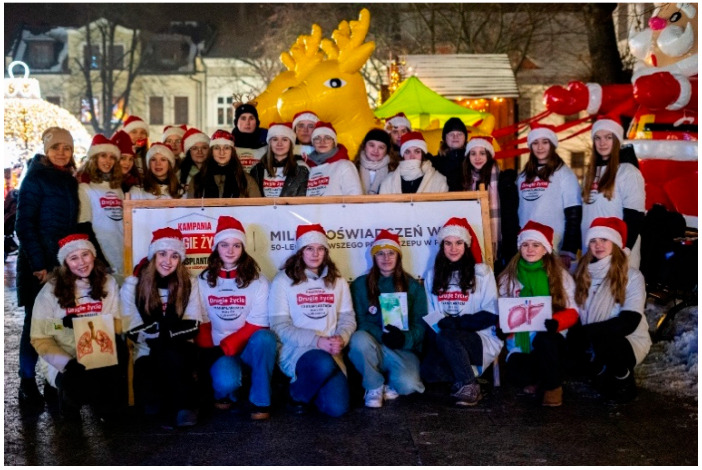
Christmas performance provided by students (source: www.drugiezycie.eu).

**Figure 4:**
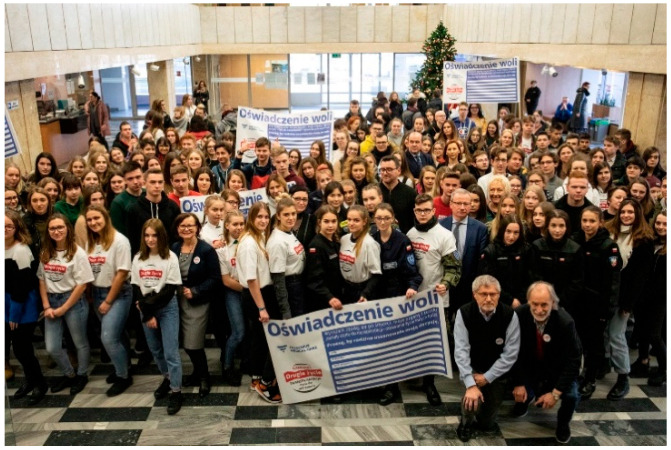
XV edition of the Second Life campaign launch (source: www.drugiezycie.eu).

**Figure 5:**
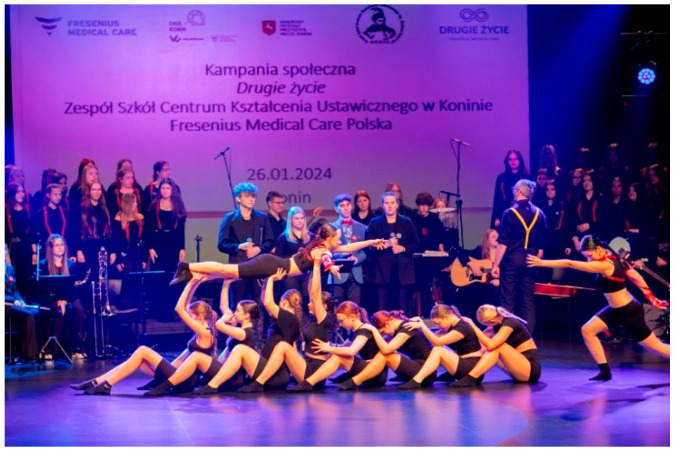
Artistic performance during the XVIth edition of the Second Life campaign (source: www.drugiezycie.eu).

**Figure 6:**
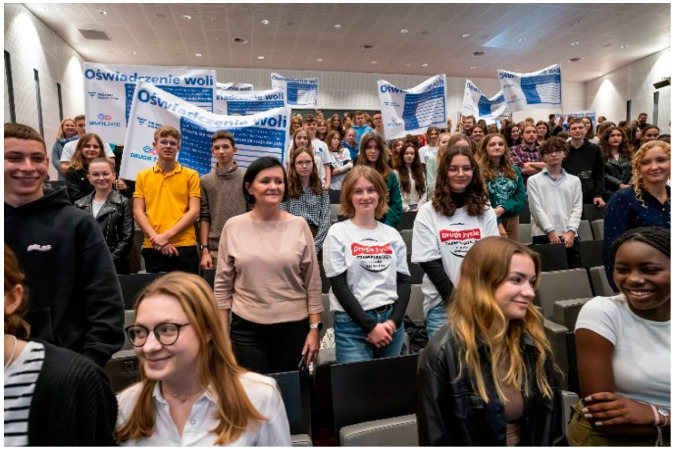
XVI edition of campaign inauguration (source: www.drugiezycie.eu).

**Figure 7:**
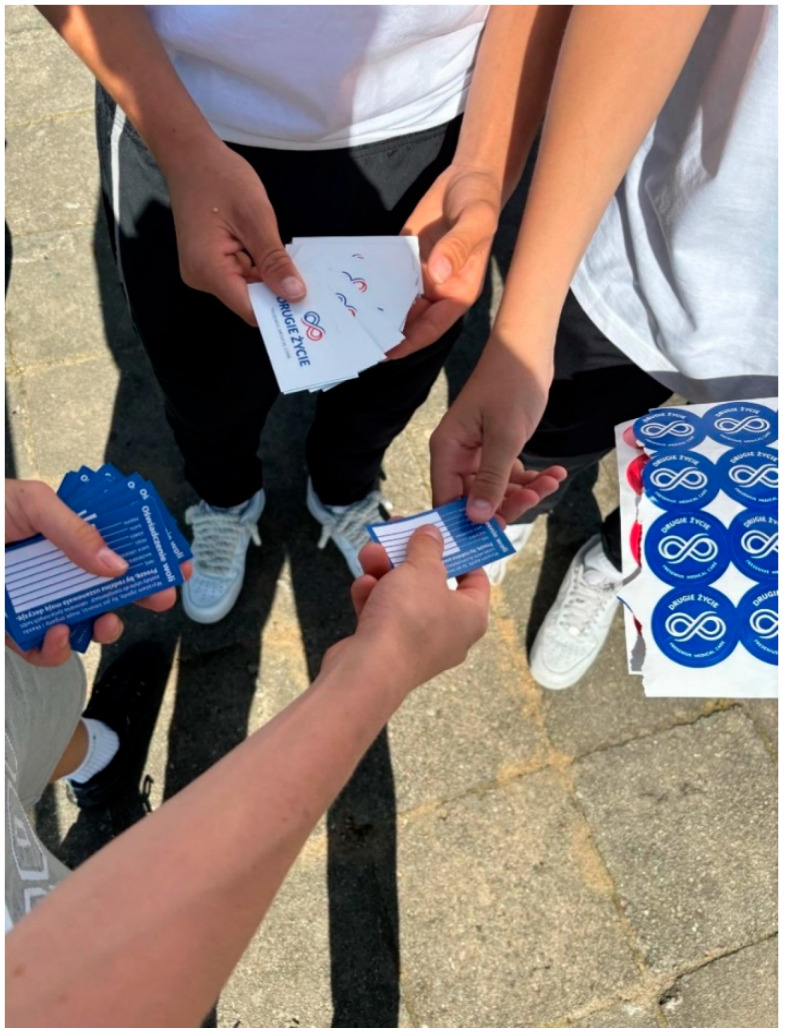
“Declaration of will” sharing on the streets (source: www.drugiezycie.eu).

**Figure 8:**
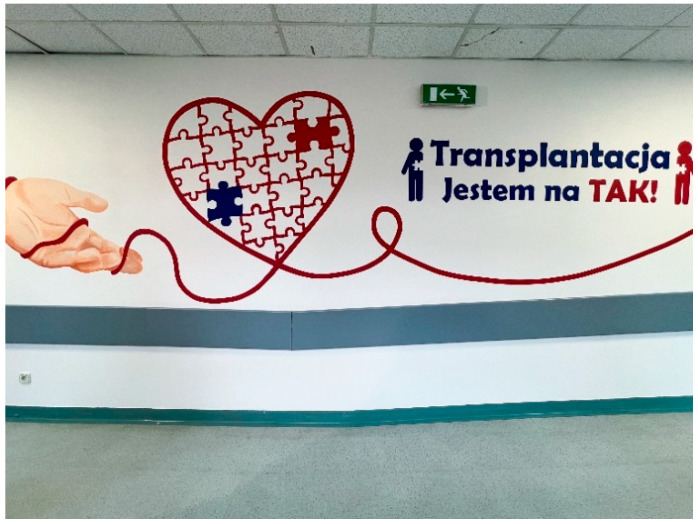
Mural painted by students in the Regional Hospital in Poznan lobby “Transplantation—I am for it” (source: www.drugiezycie.eu).

Many students prepare creative works, such as artwork displayed at school or public locations, to raise awareness about organ transplantation. These activities include setting up information booths, hosting concerts, organizing marches, writing poems, composing songs, and staging short theatrical performances (Figure 3, Figure 4, Figure 5, Figure 6 and Figure 7). Each year, there is a noticeable increase in the use of social media and modern communication tools, such as podcasts and video podcasts. The campaign has also left a lasting mark in the form of a mural, created by a student at a hospital in Poznań. The mural, located in the hall of the Regional Hospital in Poznań, was designed by 16-year-old Karolina Pieczek, a high school student from Lubasz (Wielkopolskie Voivodeship, Poland). As she explained, “The puzzle pieces on the mural symbolize how someone can give a part of themselves to save another person’s life”. The idea was transferred to the hospital walls by Pamela Herot, a graduate of the University of the Arts in Poznań (Figure 8) [9].

Students approach the topic creatively and in a language that is easily understandable, choosing communication channels that are familiar and popular among their peers. This, combined with an engaging and unobtrusive way of delivering the message, ensures that the topic of organ donation reaches thousands of people.

At the end of the campaign, each school submits a report detailing the activities they carried out over the 9 months. Based on these reports, the organizer, in collaboration with experts and transplant recipients, selects the school that demonstrated the most creativity and effectively reached the largest audience with its message.

The winning school receives a cheque, which is donated to a person or institution chosen by the students. This charitable aspect of the campaign adds another layer of meaning, reinforcing the students’ commitment to helping others.

## 5. Second Life Social Campaign in Numbers

During this period, 950 high schools across the country participated in the project, with approximately 450,000 high school students actively engaged in the campaign. These students organized a variety of activities to promote organ donation and transplantation within their social circles. They distributed nearly 1.5 million declarations of intent. Additionally, they helped raise awareness about presumed consent and educated others on the concepts of organ donation and transplantation, both from deceased and living donors. Every school, its students, and local governments played an active role in the campaign. They promoted the idea of organ donation and transplantation within their communities and on social media. As the campaign was carried out locally with media involvement, it garnered significant publicity, as documented across electronic media, the press, and printed promotional materials.

## 6. Discussion

The success of the campaign can be attributed to the freedom of expression it offers, which is particularly important for young people. This freedom makes the campaign engaging, diverse, and full of surprises year after year.

However, the freedom of speech does not negate the necessary control to ensure the message remains accurate. This control is implemented from the very beginning—during training sessions. Students are equipped with reliable knowledge and are encouraged to ask experts any questions if something is unclear. The guidance from experts ensures that students do not rely on potentially distorted information from the internet or base their messages on assumptions.

By educating young people who may not have much prior knowledge in this area, the campaign helps them enter adulthood with a solid understanding that they will continue to share, not only during the campaign but throughout their lives. The importance of educating youth has been highlighted in several studies published in recent years [10,11,12,13,14,15]. These studies suggest that a lack of knowledge about transplantation may contribute to reluctance toward organ donation within society. Cantarovich et al., based on questionnaire surveys, highlighted the openness of young people to learn about transplantation and organ donation, as well as to initiate discussions about these topics with their families and friends. According to survey results, the authors suggest that even 10-year-old students are ready to receive basic education on transplantation and organ donation [13,16,17].

At the next stage, oversight is provided by a coordinator—a teacher who acts as a mentor for the students at their school. While the coordinator does not interfere with the content or approach chosen by the students, they ensure that the message is accurate and the way the topic is presented is appropriate. The coordinator also supports students in organizing events, ensuring that these activities are conducted in collaboration with an adult.

Schools regularly report their activities to the organizer, allowing the events to be showcased on the campaign’s dedicated Facebook page and website. This ongoing communication gives the organizer the ability to monitor the accuracy and appropriateness of the students’ efforts.

The control mechanism, which respects students’ creativity, ensures that the campaign remains both educational and credible. This is one of the reasons why the campaign is supported by local governments, education boards, and religious authorities.

The freedom of speech and expression enables fresh ideas for promotion to emerge each year as new students join the campaign. Thanks to the enthusiasm and innovation of these new participants, as well as their use of new communication channels popular among young people, the campaign reaches a wide and diverse audience. The competitive aspect ensures that the activities are sustained throughout the entire school year, rather than being limited to one-time events.

The campaign’s success is driven by the boundless creativity and willingness of young people, ensuring that it has the potential to continue for many years. In our previous studies involving 569 medical students, the majority (>90%) supported organ donation from deceased family members and expressed their intention to donate their organs after death [18]. More than half of the students (54.77%) agreed to organ donation, even for non-related individuals, after death. Medical students emphasized that patients and donors (65.38%), universities (49.56%), and the media (44.64%) should be actively involved in transplantation discussions. In line with this, students stressed the urgent need for educational campaigns, citing dissatisfaction with their knowledge, which was mainly obtained through classes and seminars (89.1%) [18].

On the other hand, law students generally accepted both cadaveric and living organ donation but were less willing to accept organ donation from family members [19]. This underscores the importance of early education, particularly at the high school level [20]. In our survey of law students, 32% did not understand the difference between dialysis and transplantation, while 13% still believed that family consent for organ donation was necessary. Authors have noted a trend among law students to be overly optimistic about transplantation statistics, suggesting that awareness and understanding could be better [21].

When comparing knowledge and acceptance of organ donation between medical and technical university students, unsurprisingly, medical students had greater knowledge and a higher level of acceptance toward organ donation and transplantation [22]. Based on these findings, it seems clear that young people, particularly those from non-medical faculties, favor ongoing educational campaigns. Additionally, our assessment of attitudes towards organ donation and transplantation in the adult population of the Podlasie region of North-Eastern Poland revealed that age and education level strongly influence one’s views on organ donation [23,24,25,26,27,28]. Younger individuals and those with higher levels of education are more likely to support organ donation. Residents of larger cities are also more positively inclined toward organ donation and transplantation compared to rural inhabitants. However, rural residents, despite being open to cadaveric and living organ donation, tend to be less enthusiastic about donation when it involves family members [28]. Therefore, we emphasize the need for targeted education and awareness programs, particularly for residents of small towns and villages and for adults with primary or vocational education [27].

A study conducted in Sweden among high school and university students highlighted the deficiencies in school-based education about organ donation and the donation process. Students expressed dissatisfaction with the limited information provided by their schools on organ donation and indicated a strong interest in learning more. Interviews with students revealed the need for structured, periodic training on organ donation and transplantation [29]. The “Second Life” campaign, which has been running continuously for 17 years, stands as an example of how to fill this gap in transplant education.

Recent studies on the attitudes of Hispanic Americans in California regarding organ donation have shown the impact of outreach interventions, such as television and radio commercials and educational programs at high schools and Catholic churches. These interventions maintained awareness, significantly increased knowledge, and improved the willingness to donate organs [30,31,32,33,34]. In addition, women were found to be more likely to consent to donation than men [30,32]. Similarly, Loban et al. [35] reported that women, especially mothers and wives, were more likely to be living kidney donors. Berry et al. [30] surveyed 5444 high school students aged 15 to 20 years in California, finding that family support, being in the 11th or 12th grade, gender, religion, and the belief that Hispanic Americans are more likely to need transplants were all predictors of the intent to donate. Based on these findings, Cantarovich et al. [13] proposed incorporating organ donation and transplantation education in elementary and high school curricula.

In 2024, Herrera-Guyol and Cantarovich introduced the Connecting D.O.T.S. online platform, provided by The Transplantation Society, to educate schoolchildren globally about organ donation and transplantation. This platform includes modules for students, teachers, and parents [36]. In the Netherlands, teachers developed an interactive educational program to help lower-educated students make well-informed decisions about organ donation and transplantation [37].

In our campaign, youth served as the driving force behind educating others on organ donation and transplantation, underscoring the effectiveness of involving young people in such vital education and awareness efforts. A survey conducted among nursing students in Poland, aged 18 to 25, explored their knowledge and opinions on organ donation and transplantation [38]. Half of the participants admitted that their close relatives were unaware of their consent to donate organs after death. The authors emphasize the need for more intensive education for medical staff to address this gap. Similarly, a study of 400 university students from Northern Cyprus [39] revealed that only 27% had sufficient knowledge of organ transplantation, though two-thirds expressed positive views on organ donation. Furthermore, 37% were willing to become living donors, while 64% were open to donating their organs after death. The authors advocated for the inclusion of organ donation and transplantation education in university curricula, with further steps involving collaboration with transplant hospitals. In the more recent study, Olsacher et al. [40] recruited 1000 German citizens between 18 and 70 years via Facebook and Prolific between June and August 2022. They showed that Instagram in Germany was an underleveraged but potentially effective platform to spread the knowledge on organ donation. Another interesting study by Samin et al. [41] targeted patients and visitors aged 18 to 60 at the Outpatient Departments of Lady Reading Hospital in Peshawar, Pakistan. Among 342 individuals, the vast majority (82.18%) had not heard of Pakistan’s Organ Donation Registry, yet more than half (58.09%) expressed a willingness to donate. The study identified religion and a lack of knowledge about the laws related to organ donation as significant barriers to joining the national organ donation registry. The authors recommended increased awareness as a key strategy to promote organ donation and transplantation.

Similarly, a recent Italian study by Tattoli et al. [42] also emphasized the importance of encouraging people to register their organ donation decisions. In contrast, Moonajilin et al. [43] investigated understanding, attitudes, and intent to donate in Bangladesh’s adult population. They stressed the need for national education campaigns and awareness-raising events to increase public knowledge about organ donation and transplantation. Despite adults’ positive attitudes toward organ donation, the study found that many lacked adequate knowledge. Notably, certain ethnic groups, such as the Roma, are more sensitized to living donor transplantation [44,45], suggesting that targeted campaigns are crucial to increasing awareness and donation rates.

## 7. Limitations

Lack of knowledge remains the primary barrier to organ donation and transplantation, though religious beliefs and education also significantly influence donation rates. In the case of living donation, trust and transparency throughout all procedures are crucial to minimize the risks of coercion, prevent organ trafficking, and ensure that the donor’s decision is fully informed and voluntary.

Furthermore, recent research by Kaminski et al. [46] highlights that a social campaign promoting organ donation, along with legislative changes in transplantation-related laws and improvements in healthcare professionals’ training, could enhance working conditions for transplant coordinators in Poland. However, the long-term impact of social campaigns on raising awareness and fostering acceptance, ultimately expanding the donor pool and increasing the number of transplants, remains an unresolved issue. Despite 17 years of our campaigns, much work still lies ahead in this field.

## 8. Conclusions

Social media and campaigns play a crucial role in raising and enhancing awareness about organ transplantation in society. The data from the Second Life campaign was presented orally at The Transplantation Congress in Istanbul, Turkey, from 22 to 25 September 2024, and the abstract was published in Transplantation [47]. During the oral presentation, the discussion was lively and productive, prompting us to share our experiences in an open-access publication.

High school students were highly motivated, creative, and their contributions to the campaign were well-received by the public. Each year, the number of participating schools has increased, further strengthening the rationale for continuing these efforts and expanding the campaign’s reach. Additionally, nephrologists, cardiologists, and transplant physicians have become more devoted to the idea and are increasingly involved in this project.

Although it is not yet possible to directly assess whether this campaign has contributed to the increase in the number of organ transplants, raising awareness, educating the public, and fostering a positive attitude toward organ donation are vital steps. In 2024, Poland reached a record high in the number of transplants, with a total of 2152 procedures, including 1130 kidney transplants, 615 liver transplants, 202 heart transplants, 147 lung transplants, 10 pancreas transplants, and 46 combined transplants, according to the latest report from Poltransplant [48].

## Figures and Tables

**Table 1:** Milestones of each campaign and the activities.

Date	Activity
September	School registration opens. Schools are selected based on submitted applications (30–32 schools per edition). Information is provided to the selected schools.
October–November	Campaign inauguration Basics of transplantation pieces of training conducted in selected schools (remotely or in-place)
November–May	Space for students’ actions, e.g.,: Artistic performances promoting transplantation awareness Visits to workplaces and institutions Participation in events organized by external stakeholders, such as motorbike meetings, music festivals, local festivals, etc. Collaboration with local churches Street performances (marches, street theater, etc.)
June	Selection of the best activities (conducted by the organizers and patrons) Award ceremony Closing of the campaign edition.

## Data Availability

The data presented in this study are available at https://firebasestorage.googleapis.com/v0/b/drugie-zycie.appspot.com/o/files%2F3IpACrcqXayTyGuI.pdf?alt=media&token=987793ce-cf4f-43a9-9ac5-e60231cf75bc (accessed on 1 February 2025).
